# Research on Yield Prediction Model Driven by Mechanism and Data Fusion

**DOI:** 10.3390/s25061946

**Published:** 2025-03-20

**Authors:** Xin Meng, Xingyu Liu, Hancong Duan, Ze Hu, Min Wang

**Affiliations:** 1 School of Electrical Information, Southwest Petroleum University, Chengdu 610500, China; 202222000110@stu.swpu.edu.cn (X.M.);; 2 School of Computer, University of Electronic Science and Technology of China, Chengdu 611731, China

**Keywords:** time-series forecasting, mechanistic model, production forecasting, transformer

## Abstract

Existing production forecasting methods often suffer from limited predictive accuracy due to their reliance on single-source data and the insufficient incorporation of physical principles. To address these challenges, this study proposes a mechanism–data fusion production forecasting model that integrates mechanistic model outputs with data-driven learning techniques. The proposed method first establishes a three-phase-separator mechanistic model to generate physics-informed simulation data. Then, a Global–Local Branch Prediction Model is designed to enhance both long-term trend estimation and local feature capture in a production time series. The mechanistic model data are incorporated as constraints into the prediction framework, effectively guiding the learning process and improving forecast accuracy. Experimental results on real-world oilfield data demonstrate that the proposed model outperforms state-of-the-art methods such as Autoformer and DLinear. Specifically, under the mechanism-based approach, the Global–Local Branching Prediction Model reduces MSE by 0.0100, MAE by 0.0501, and RSE by 1.40% compared to Autoformer and achieves improvements of 0.0080 in MSE, 0.0093 in MAE, and 0.48% in RSE over DLinear. The results confirm that integrating mechanistic constraints significantly enhances prediction performance, making the proposed model a robust and technologically superior solution for production forecasting in petroleum engineering.

## 1. Introduction

Accurate oil production forecasting is essential for optimizing reservoir management, well performance evaluation, and production planning. However, traditional forecasting methods often face challenges in handling complex reservoir dynamics, production fluctuations, and external influences. Existing approaches primarily fall into two categories: data-driven models and mechanistic models, each with inherent limitations.

In recent years, most oil production forecasting methods have directly relied on operational data [1], ranging from traditional statistical approaches to advanced machine learning models [2]. Among the most popular methods are multi-cycle models, long short-term memory (LSTM) networks, Support Vector Machines (SVMs), and the Autoregressive Integrated Moving Average (ARIMA) model. While these methods excel at identifying trends and short-term fluctuations, they often struggle with nonlinear reservoir behavior, varying well conditions, and long-term forecasting accuracy. For instance, Wei B et al. [3] developed a three-dimensional nonlinear flow model for multiphase production prediction, incorporating imbibition effects to improve forecasting in fractured reservoirs. Liu W. et al. [4] introduced an EEMD-based LSTM approach, integrating empirical mode decomposition with deep learning to enhance prediction accuracy in Chinese oilfields. Qiao Y. et al. [5] employed a Particle Swarm Optimization (PSO)-based Least Squares Support Vector Machine (LSSVM) model to improve convergence speed and forecasting reliability. Rajni R. et al. [6] demonstrated the applicability of ARIMA models in renewable energy production forecasting, highlighting their generalization ability across various energy sectors. While these methods effectively capture historical trends, they suffer from limited interpretability, data dependency, and poor adaptability to changing reservoir conditions. More importantly, they fail to incorporate physical constraints, making them unreliable in cases where historical data alone are insufficient.

Mechanistic models offer a physics-based perspective, leveraging fluid dynamics, thermodynamic principles, and reservoir characteristics to predict production behavior. These models simulate real-world conditions, making them highly interpretable and theoretically robust. Notable studies include those by Eshkalak et al. [7], who developed a hydraulically fractured well model that accounted for nonlinear pressure-dependent behaviors. Ali et al. [8] provided a comprehensive classification of multiphase flow models, discussing their applications and highlighting areas requiring further research. Zhao et al. [9] conducted high-pressure, high-temperature experiments to analyze oil–water interactions in tight reservoirs. Jiang et al. [10] built a coupled fluid flow and geomechanics model, incorporating fracture interactions for more precise shale gas production forecasting. Despite their strengths, mechanistic models require precise input parameters and extensive computational resources and often fail to adapt to changing field conditions. More critically, they lack the ability to learn from historical production data, limiting their predictive flexibility.

This study combines historical data, which provide a temporal perspective on oil production trends, with mechanical data, which offer insights into the underlying physical processes affecting production efficiency. The integration of these two types of data aims to improve the accuracy and robustness of the forecasting model. Therefore, we propose a prediction method that integrates mechanical model data. By developing a mechanistic model for a three-phase separator [11], we can obtain mechanistic data, which, when fused with traditional production data (such as temperature and pressure), form a multi-dimensional constraint for the forecasting model. To achieve long-term high-accuracy forecasting, we used a Global–Local Branch Prediction Model, significantly improving prediction accuracy while simplifying the model size. Its application to a real-world field setting shows that the proposed method outperforms other forecasting models, demonstrating higher feasibility and effectiveness.

The paper is structured as follows: Section 2 reviews related work; Section 3 introduces the mechanism model, Global–Local Branch Prediction, and Mechanism–Data Fusion Prediction Model; Section 4 presents the experimental results; and Section 5 discusses conclusions and future research directions.

## 2. Overall Framework

**Figure 1:**
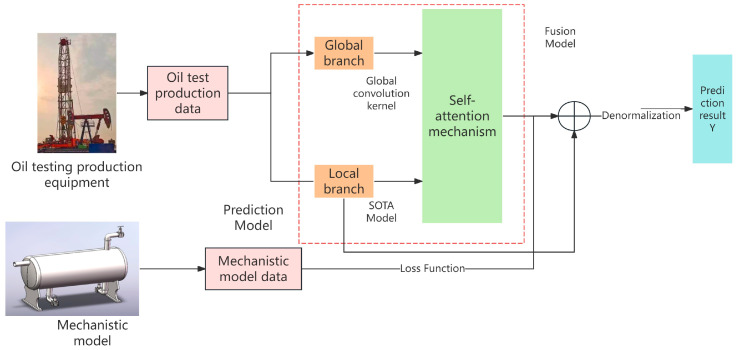
Mechanism–Data Fusion Prediction Model.

This paper introduces a production forecasting model that integrates mechanistic model data, combining idealized equipment physics modeling data with actual historical data from oil well equipment sensors. By unifying these two data sources, the proposed method aims to improve the accuracy and reliability of production prediction. The following are the innovations of this paper. Figure 1 illustrates the overall framework, which includes building a mechanical equipment model, designing a time-series prediction algorithm, and fusing mechanistic model data.

1. Mechanistic Equipment Modeling

A mechanistic model of oil well equipment was developed to generate mechanistic model data. Specifically, we mathematically analyzed and modeled the fluid behavior within a three-phase separator. Feedback control and PID fuzzy control strategies were applied to simulate ideal pressure and temperature profiles, thereby producing mechanistic model outputs.

2. Global–Local Branching Forecast Model

To capture both long-term dependencies and local patterns in the production time series, the forecasting model was divided into a global branch and a local branch. The global branch leverages global convolutional kernels with three distinct parameterization approaches—multi-scale, frequency-domain, and Legendre-domain approaches—to extract broad temporal features. Meanwhile, the local branch employs a state-of-the-art (SOTA) framework to capture localized data characteristics. The outputs from both branches were combined via a self-attention module, followed by a linear transformation. Finally, a denormalization process was conducted to obtain the production forecast.

3. Fusion of Mechanistic Model Data

To fully exploit the value of mechanistic model outputs, we encoded these data over time and incorporated them as fixed constraints within the loss function. This additional constraint enhanced the model’s learning capacity, narrowing the gap between the predicted and actual production values. As a result, the overall forecasting accuracy for oil well production was significantly improved.

## 3. Methodology

### 3.1. Mechanistic Model Design

#### 3.1.1. Mathematical Model of Three-Phase Separator

During oil and gas gathering, the mixture of oil and gas must be separated into specialized equipment. According to equilibrium principles and leveraging the mechanisms of oil–gas separation, mechanistic methods can be employed to divide the mixture into gaseous and liquid phases [12]. In general, devices designed for oil–gas separation are known as oil–gas separators [13].

A horizontal three-phase separator, commonly used in oilfields, consists of four main sections: the inlet distribution zone, liquid-collecting zone, gravity settling zone, and demisting zone [14]. When oil well fluids enter, they first collide with an inlet baffle, causing an initial gas–liquid separation. The pre-separated liquid then falls into the liquid-collecting zone, allowing oil to rise and water to settle. A liquid conduit is often installed to guide water droplets below the oil–water interface for better separation.

The oil layer overflows a weir plate into an oil chamber, where a liquid-level control valve maintains the oil level. Similarly, the water layer is discharged through another control valve. Pre-separated gas enters the gravity settling zone, where larger liquid droplets separate out before passing through a demister. A pressure control valve regulates gas discharge, maintaining consistent separator pressure. The gas–liquid interface is typically half-full but may vary based on the separation requirements.

**Figure 2:**
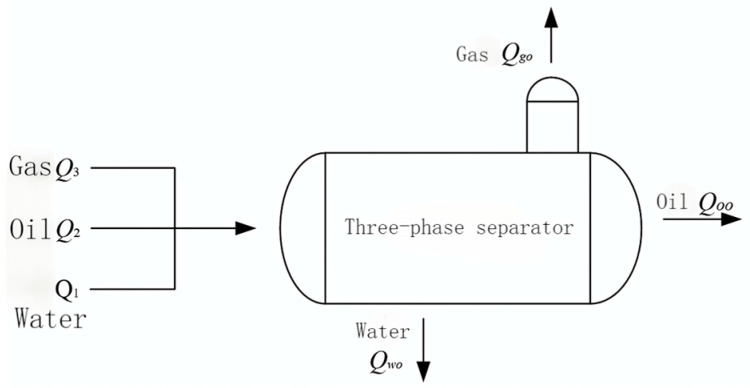
Material balance diagram of three-phase separator.

**Figure 3:**
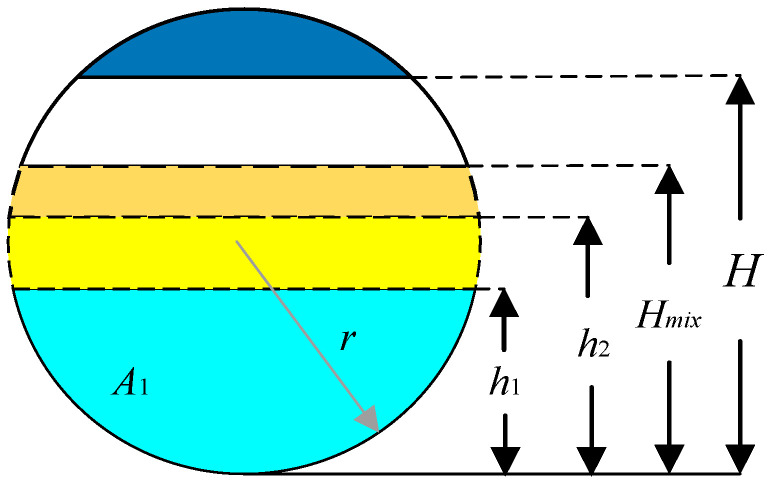
Longitudinal section of a three-phase separator (right side).

**Figure 4:**
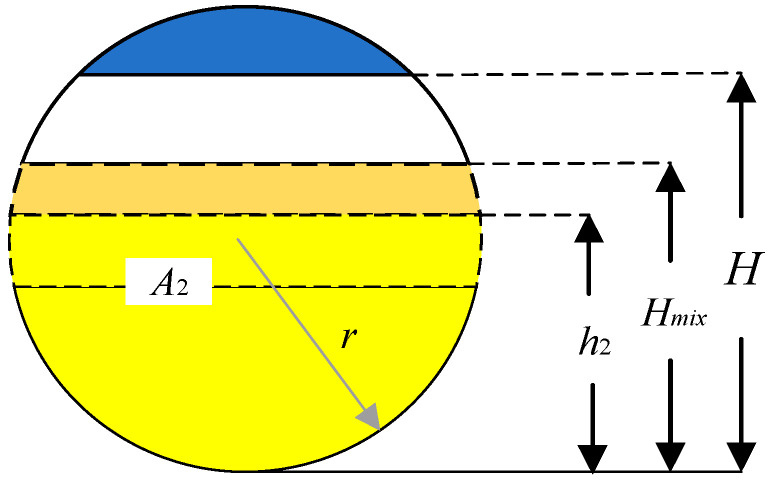
Longitudinal section of a three-phase separator (left side).

**Figure 5:**
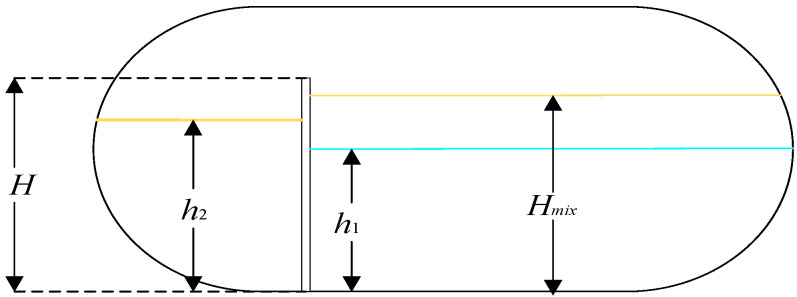
Transverse section of three-phase separator.

The simulation of the three-phase separator is needed to calculate the difference in the liquid level and gas pressure change in the three-phase separator based on the known operating parameters, such as the separator’s structure size, the water content of the inlet crude oil, and the inlet flow rate [15]. The material balance of the three-phase separator is shown in Figure 2. The simulation modeling treats the horizontal three-phase separator as a cylindrical container and controls its input variables, where $u_{1}$, $u_{2}$, and $u_{3}$ denote the opening degrees $\mu_{i}$ of the water discharge valve, oil discharge valve, and gas discharge valve, respectively. The three-phase separator’s output variables include the oil–water interface height $H_{\mathrm{mix}}$, the liquid level $h_{2}$ in the oil chamber, and the vessel’s internal gas pressure P. The longitudinal and transverse sections of the three-phase separator are shown in Figure 3, Figure 4 and Figure 5.

**Figure 6:**
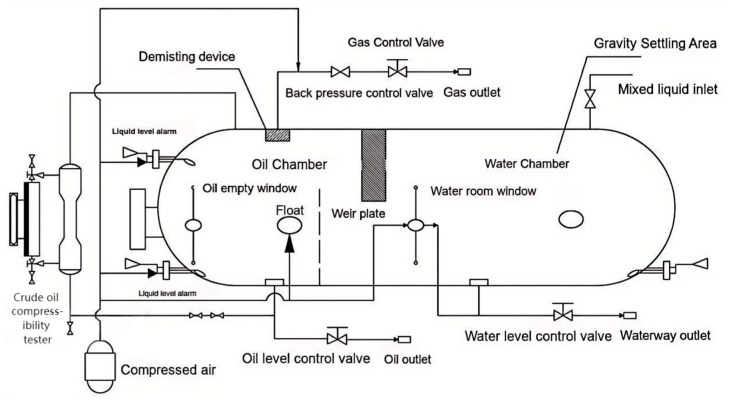
Schematic diagram of horizontal three-phase separator structure.

The three-phase separator separates gas, oily wastewater, and water-containing crude oil, following the law of mass conservation. The material balance model is shown in Figure 6. The material balance equation governing water flow is as follows:

(1) $$A_{1}\frac{dh_{1}}{\mathrm{dt}}=Q_{1}-Q_{\mathrm{wo}}$$

where $A_{1}$ is the cross-sectional area of water below the oil–water interface, $h_{1}$ is the water level, $Q_{1}$ is the water inlet volume flow rate, and $Q_{\mathrm{wo}}$ is the volume flow rate of water flowing out of the separator.

Assuming the presence of a linear valve and ignoring the density changes, the Bernoulli equation simplifies this to the following:

(2) $$P_{1}+\frac{1}{2}\rho {v_{1}}^{2}=P_{2}+\frac{1}{2}\rho {v_{2}}^{2}$$

where $P_{1}$ is the pressure before the valve, $v_{1}$ is the flow rate before opening, $v_{2}$ is the flow rate at the moment of opening, and ρ is the density of the liquid passing through the valve.

Given that $v_{1}=0$, the pressure difference across the water outlet valve is as follows:

(3) $$P_{1}=P_{2\;}+\frac{1}{2}\rho {v_{2}}^{2}$$

Then, the pressure difference between the two ends of the water outlet valve is as follows:

(4) $$∆P_{w}=\frac{1}{2}\rho {v_{2}}^{2}$$

From the flow formula Q=SV, where Q is the flow, S is the cross-sectional area, V is the water flow rate, we obtain the following:

(5) $$Q_{\mathrm{wo}}=K_{v1}S_{1}\sqrt{\frac{2∆P_{w}}{\rho_{1}}}$$

where $K_{v1}$ is the flow coefficient, $∆P_{w}$ is the pressure difference across the outlet valve, $S_{1}$ is the cross-sectional area of the outlet valve, and $\rho_{1}$ is the density of water. The relationship between valve opening $\mu_{i}$ and flow coefficient is shown as follows:

(6) $$K_{\mathrm{vi}}=K_{i}\mu_{i}$$

where $K_{i}$ is a proportional constant and $\mu_{i}$ is the opening of the valve, usually expressed as a percentage. In actual engineering, the opening range of linear valves is determined based on the process conditions, fluid characteristics, and regulation system requirements [16]. The pressure difference across the valve is as follows:

(7) $$∆P_{w}=P+\rho_{2}g(H-h_{1})+\rho_{1}gh_{1}-P_{\mathrm{wo}}$$

where P is the gas pressure, g is gravity, $P_{\mathrm{wo}}$ is the outlet pressure, and H is the height. The water level control equation is as follows:

(8) $$A_{1}\frac{dh_{1}}{\mathrm{dt}}=Q_{1}-K_{1}\mu_{1}S_{1}\sqrt{\frac{2[P+\rho_{2}g(H-h_{1})+\rho_{1}gh_{1}-P_{\mathrm{wo}}]}{\rho_{1}}}$$

Similarly, the oil level control equation is as follows:

(9) $$A_{2}\frac{dh_{2}}{\mathrm{dt}}=Q_{2}-K_{2}\mu_{2}S_{2}\sqrt{\frac{2(P+\rho_{2}gh_{2}-P_{\mathrm{oo}})}{\rho_{2}}}$$

where $A_{2}$ is the oil chamber cross-sectional area, $h_{2}$ is the oil chamber level, $Q_{2}$ is the oil inlet volume flow rate, $K_{2}$ is the slope corresponding to the flow characteristic curve of the oil outlet valve, $\mu_{2}$ is the opening of the oil outlet valve, $S_{2}$ is the cross-sectional area of the oil outlet valve, and $P_{\mathrm{oo}}$ is the outlet pressure.

For gas-phase dynamics, the material balance equation is as follows:

(10) $$\frac{\mathrm{dn}}{\mathrm{dt}}=Q_{3}-Q_{\mathrm{go}}$$

where n is the number of moles of gas, $Q_{\mathrm{go}}$ is the gas outlet flow rate, and $Q_{3}$ is the molar flow rate.

(11) $$Q_{\mathrm{go}}=K_{3}\mu_{3}S_{3}\frac{P}{\mathrm{RT}}\sqrt{\frac{P-P_{\mathrm{go}}}{M_{w}\mathrm{RT}}}$$

where $K_{3}$ is the coefficient, $\mu_{3}$ is the opening of the outlet valve, $P_{\mathrm{go}}$ is the outlet pressure of the outlet valve, R is the gas constant, T is the separator temperature, $S_{3}$ is the cross-sectional area, and $M_{w}$ is the average molecular mass. Using the ideal gas law, the pressure control equation is given as follows:

(12) $$\frac{\mathrm{dP}}{\mathrm{dt}}=\frac{\mathrm{RT}}{V}\;({\;Q}_{3}-K_{3}\mu_{3}S_{3}\frac{P}{\mathrm{RT}}\sqrt{\frac{P-P_{\mathrm{go}}}{M_{w}\mathrm{RT}}})$$

where V is the gas volume, given by the following:

(13) $$V=V_{0}-V_{1}$$

where $V_{0}$ is the separator volume and $V_{1}$ is the liquid volume related to liquid level h.

(14) $$V_{1}=L[(h-r)\sqrt{r^{2}-{(h-r)}^{2}}+r^{2}\arcsin(\frac{h-r}{r})+\frac{\pi r^{2}}{2}]$$

where r is the separator radius and L is the separator length.

The parameters required to establish the dynamic model of the three-phase separator are shown in Table 1.

#### 3.1.2. MPC Controller Design

In the dynamic model simulation of the three-phase separator, valves act as controlled elements. The input variable is the initial liquid level height, the control variable is the valve opening degree, and the output variables include the adjusted oil-phase liquid level, water-phase liquid level, and pressure [17]. When inlet flow increases, the liquid level rises, requiring a larger valve opening to increase the outlet flow, and vice versa. This system operates under a negative feedback mechanism, where the liquid level height serves as a feedback signal to regulate valve opening, ensuring stability.

For water-level control, assuming a steady state and a linear discharge valve, the separation interface height $h_{1}$ is proportional to the water discharge flow rate $Q_{\mathrm{wo}}$:

(15) $$k=\frac{Q_{\mathrm{wo}}}{h_{1}}$$

where k is a proportional constant. This can be substituted into the mass balance equation as follows:

(16) $$\frac{dh_{1}}{\mathrm{dt}}=\frac{1}{A_{1}}(Q_{1}-kh_{1})$$

**Figure 7:**
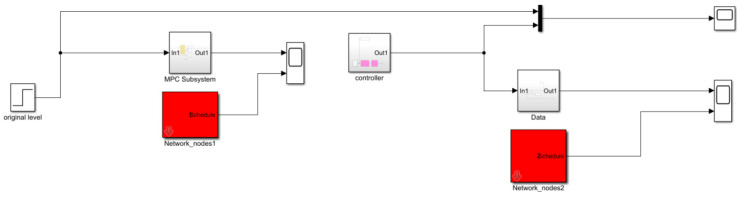
Liquid-level feedback control model.

**Figure 8:**
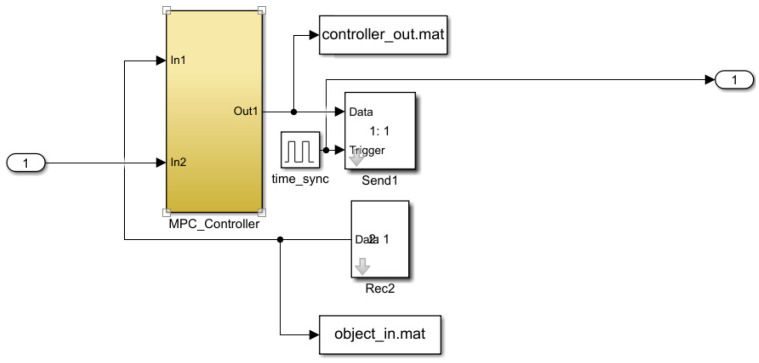
MPC control module.

**Figure 9:**
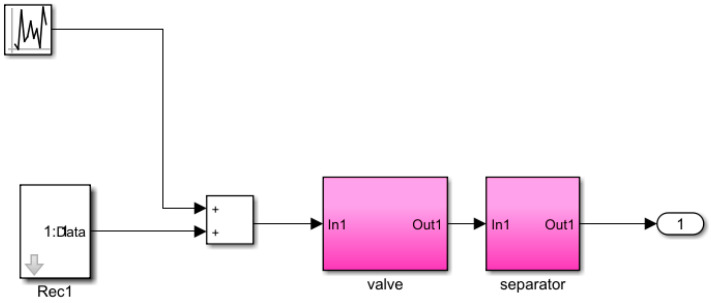
Valve module.

**Figure 10:**
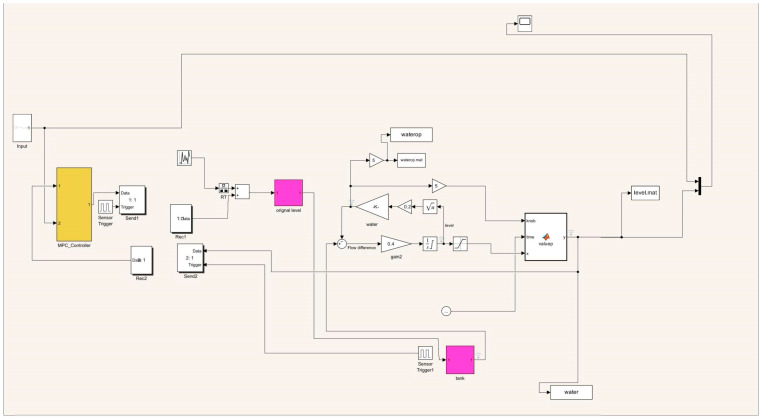
Liquid-level control model.

**Figure 11:**
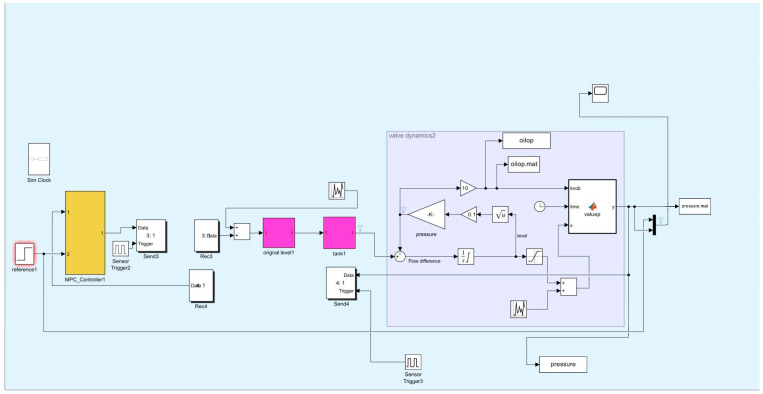
Pressure control model.

This is then simplified to a first-order time-delay differential equation, and using the Laplace transform, the control system transfer function is as follows:

(17) $$\frac{H_{1}\left(s\right)}{Q_{1}\left(s\right)}=\frac{1}{A_{1}(s+k)}$$

where s refers to the complex variable in the Laplace transform. The feedback control model is simulated in Simulink (version R2023b), as shown in Figure 7, Figure 8, Figure 9, Figure 10 and Figure 11.

### 3.2. Mechanism–Data Fusion Prediction Model Design

#### 3.2.1. Forecasting Model Formulation

To predict oil production more accurately, we propose the use of a Mechanism–Data Fusion Forecasting Model, which integrates statistical learning with mechanistic constraints. The forecasting model is defined as follows:

The time-series dataset $D={\left\{X_{t},Y_{t}\right\}}_{t=1}^{T}$ is used, where $X_{t}\in R^{n}$ is the input feature vector at time t, including temperature, pressure, and mechanistic model outputs. $Y_{t}$ is the target variable, representing the oil production rate at time t.

We define the forecasting function as follows:

(18) $${\hat{Y}}_{t+1}=f_{\theta }\left(X_{t}\right)$$

where $f_{\theta }$ is the prediction model parameterized by θ. To enhance long-term predictive accuracy, we recommend using a Global–Local Branching Prediction Model as follows:

(19) $${\hat{Y}}_{t+1}=W_{g}G\left(X_{t}\right)+W_{l}L\left(X_{t}\right)$$

where $G\left(X_{t}\right)$ represents the global branch, capturing long-term production trends using a convolutional operator. $L\left(X_{t}\right)$ represents the local branch, leveraging self-attention mechanisms for short-term pattern recognition. $W_{g}$ and $W_{l}$ are learnable weights that balance the contributions of both branches.

The final prediction is obtained through mechanism-constrained optimization, and the design of the loss function is described in Section 3.2.3.

#### 3.2.2. Global–Local Branch Prediction Model

Transformer-based models have emerged as powerful tools for time-series forecasting [18]. However, these models often struggle when dealing with long input sequences. One key issue is their inability to capture the long-range dependencies present in time-series data. Another is that extended input sequences tend to lead to larger model sizes and higher computational complexity. To address these limitations, this study introduces a novel Global–Local architecture, which combines a low-complexity global convolution branch to handle long input sequences [19] with a local Transformer-based branch for capturing shorter and more recent signals. This architecture leverages a global convolution kernel [20] within a cohesive framework augmented by SOTA models for improved long-horizon forecasting.

The global branch primarily utilizes a global convolution kernel, as it excels at capturing long-range dependencies [21]. Specifically, three different parameterization methods are applied: multi-scale parameterization, frequency-domain parameterization, and Legendre-domain parameterization. Meanwhile, the local branch adopts a SOTA framework to extract local features effectively.

An input sequence $u\in R^{n\times d}$, a learnable global kernel $k\in R^{n\times d}$, and output $y\in R^{n\times d}$ are used. The global convolution kernel is expressed as follows:

(20) y=u∗k

where ∗ is the convolution operator. Although the complexity of the global convolution operation is $O(N^{2})$, it can be quickly implemented using the fast Fourier transform, which has a complexity of O(NlogN), resulting in the following:

(21) $$u*k=F^{-1}(F(u)\cdot F(k))$$

A global convolution kernel that scales its parameters linearly with the sequence length poses significant challenges for efficient and effective feature extraction [22]. To ensure that the number of parameters increases only linearly with the sequence length, a multi-scale parameterized kernel is constructed by assembling a series of progressively larger sub-kernels. Each sub-kernel is upsampled from the same base set of parameters via interpolation techniques. In addition, these sub-kernels are combined through a weighted scheme in which the weights decay progressively. This strategy provides a beneficial inductive bias for modeling extended sequences, ultimately improving performance. Using a kernel $k_{msk}$, parameterized through multi-scale sub-kernels, the global convolution model is defined as follows:

(22) $$y=F^{-1}(F(u)\cdot F(k_{msk}))$$

Time–frequency transformation offers an alternative approach to parameterizing the global convolution kernel, particularly for time-series data that exhibit noticeable biases in both domains [23]. Instead of generating kernels in the time domain—like in the multi-scale parameterization approach—frequency-domain parameterization uses a compact representation of the time series in the frequency domain [24]. Formally, we denote the frequency-domain kernel as $k_{freq}\in C^{m\times d}$. To ensure a sub-linear kernel size, we constrained m≪n, where n is the original sequence length. The process for the global convolution model with a frequency-domain parameterized kernel is defined as follows:

(23) $$y=F^{-1}(F(u)\cdot F(k_{freq}))$$

Using state-space models to process sequence data provides a new perspective beyond the kernel parameterization of Fourier transform [25]. Following the process of the state-space model, $x_{k}=Ax_{k-1}+Bu_{k}$, $y_{k}=Cx_{k}+Du_{k}$, where $u_{k}\in R^{d}$ is the input signal at time step k, $x_{k}\in R^{d}$ is the hidden state of the state-space model, and $y\in R^{d}$ is the output. The state transition matrix A and the input matrix B are defined as follows:

(24) $$A_{nk}=(2n+1)\left\{\begin{array}{c} (-1{)}^{n-k},\mathrm{if}\;k\leq n\\ 1,\mathrm{if}\;k\geq n \end{array}\right.\;B_{n}=(2n+1)(-1{)}^{n}$$

Here, the matrices $A\in R^{d\times d}$ and $B\in R^{d\times 1}$ are derived using the Legendre (LegT) metric, which assigns uniform weights to recent historical data. $C\in R^{1\times d}$ and $D\in R^{1\times 1}$ are the output matrices. Repeated computations of the state-space model can be quickly computed using convolutions. The transformation matrices A, B, and C are all predefined, so the kernel K can be computed in advance.

(25) $$y=u*K,K=\left(CB,CAB,\cdots ,CA^{N-1}B\right)$$

The transformation matrix A is derived from the Legendre polynomials [26], so u∗K can be interpreted as the projection of the original signal from time space to Legendre space. This process is similar to projecting a signal into frequency space using the Fourier transform method. Using the Legendre metric, the projection of the signal from time space to Legendre space is expressed as $\bar{u}=LegT.Project(u)$, and the reconstruction of the signal from Legendre space to time space is expressed as $u=LegT.Reconstruct(\bar{u})$. Using a kernel $K_{leg}\in R^{m\times d}$, where m≪n, the process of the global convolution model using the Legendre metric is defined as follows:

(26) $$y=\mathrm{legt}.\mathrm{Reconstruct}\left(\mathrm{legt}.\mathrm{Project}\;(u)*K_{\mathrm{leg}\;}\right)$$

**Figure 12:**
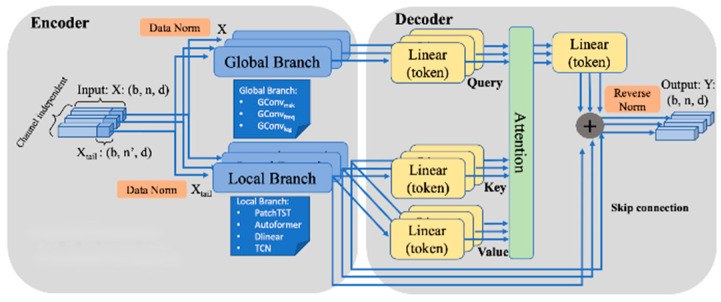
Global–Local Branch Prediction frame.

The Global–Local Branch Prediction Model involves passing the input data through two independent branches simultaneously, each of which is specially designed to capture and extract different types of local and global information. The decoder module integrates and merges these two types of information to maximize their complementarity. The overall framework of the prediction model is shown in Figure 12.

There are two parallel branches within the encoder. Specifically, the upper branch aims to extract the global information $z_{global}$ of the entire sequence $X\in R^{N\times d}$, and the complexity of the entire sequence is sub-linear with the sequence length. In contrast, the lower branch focuses on capturing the nearest local information $z_{local}$ of the tail sequence $X_{tail}\in R^{N^{\prime }\times d}$, which is the dependency between nearby time nodes. At the same time, the tail fragment of the sequence $X_{tail}\in R^{N^{\prime }\times d}(N^{\prime }<N)$ is fed to the transformer branch to reduce the overall complexity without sacrificing prediction accuracy.

(27) $$\begin{array}{c} z_{\mathrm{global}\;}={\mathrm{Branch}}_{\mathrm{global}\;}(X)\\ z_{\mathrm{local}\;}={\mathrm{Branch}}_{\mathrm{local}\;}\left(X_{\mathrm{tail}}\right) \end{array}$$

In order to improve the utilization of global and local information, the global information ($z_{global}$) and local information ($z_{local}$) are mapped to a hidden dimension at the token level. Then, the global information is used as the query (q), and local information is used as the key (k) and value (v). They are put into the cross-attention module, which can effectively integrate the global and local information. The output of the attention module is linearly transformed, added to the output of the local branch, and then denormalized to obtain the final output Y.

(28) $$\begin{array}{c} q=\mathrm{MLP}\left(z_{\mathrm{global}}\right)\\ k=\mathrm{MLP}\left(z_{\mathrm{local}}\right)\\ v=\mathrm{MLP}\left(z_{\mathrm{local}}\right) \end{array}$$

(29) $$\mathrm{Atten}(q,k,v)=\mathrm{Softmax}\left(\frac{qk^{⊺}}{\sqrt{d_{q}}}\right)$$

Among them, $qk^{⊺}$ represents the attention matrix, $d_{q}$ represents the dimension of q, and v represents the v matrix that needs to be weighed. These scores determine the weight of each input element when forming the output. The scores are normalized by the softmax function to ensure that the sum is one. The scores are interpreted as weights to obtain the output of the decoder.

First, the mean and standard deviation $x_{k}^{\left(i\right)}\in R^{T}$ are calculated for each input data instance:

(30) $$\begin{array}{c} E_{t}\left[x_{kt}^{\left(i\right)}\right]=\frac{1}{T}\sum_{j=1}^{T}\;x_{kj}^{\left(i\right)}\\ \mathrm{Var}\left[x_{kt}^{\left(i\right)}\right]=\frac{1}{T}\sum_{j=1}^{T}\;{\left(x_{kj}^{\left(i\right)}-E_{t}\left[x_{kt}^{\left(i\right)}\right]\right)}^{2} \end{array}$$

Using these statistics, the input data are normalized using a learnable affine parameter vector $\gamma ,\beta \in R^{K}$:

(31) $${\overset{ˆ}{x}}_{kt}^{\left(i\right)}=\gamma_{k}\left(\frac{x_{kt}^{\left(i\right)}-E_{t}\left[x_{kt}^{\left(i\right)}\right]}{\sqrt{\mathrm{Var}\left[x_{kt}^{\left(i\right)}\right]+\epsilon }}\right)+\beta_{k}$$

The normalized data are then fed into the model for prediction. Finally, the normalization process is reversed using the inverse of the initial normalization to obtain the prediction result.

#### 3.2.3. Mechanism–Data Fusion Method

In this study, mechanistic model data are incorporated into the loss function as an additional fixed constraint [27] by introducing a regularization term into the original loss function. The core idea is to leverage the physical and chemical principles embedded in the mechanistic model to guide and optimize the data-fusion process [28], thereby enhancing both the accuracy and reliability of the results.

Based on two datasets—one originating from field measurements at the oil well and another derived from the mechanistic model—the loss function was designed to consider errors from both datasets. In doing so, the model was guided to learn the shared features of these two data sources simultaneously. The designed loss function is shown as follows:

(32) $$L_{com}=\alpha L_{sensor}+\beta L_{model}$$

$L_{sensor}$ is a loss term based on measured data, and $L_{model}$ is a loss term based on mechanism model data using the mean square error (MSE). α and β are weight coefficients used to balance the contribution of the two loss terms, which are hyperparameters between 0 and 1.

Then, this total loss function is added to the original loss function as an additional constraint term to form a new loss function:

(33) $$L_{total}=MSE+MAE+RSE+\lambda_{\mathrm{mech}}\times L_{com}$$

Among them, $\lambda_{mech}$ is a hyperparameter used to adjust the weight of the mechanism model loss in the total loss.

## 4. Mechanism–Data Fusion Prediction Model Verification

### 4.1. Data Processing

To filter the data needed for the model, we used time-series data collected every second from an oil well, integrating 86,300 data points from each device every day. These data points reflect the continuous time changes in production parameters and are, therefore, critical for capturing dynamic trends and dependencies over time. The structure of the dataset is a multi-feature time series, where each observation consists of a timestamp and multiple sensor readings. The specific time-series features used in this model are shown in Table 2.

The mechanism model outputs, derived from the three-phase separator simulation, also follow a time-series format, capturing the separator’s physical response to production changes. These outputs, along with historical sensor readings, serve as model inputs. Gas production is used as the target data; the mechanism model data in Table 3 and the historical data in Table 2, except gas production, are input into the prediction model as characteristic data.

The measured data of wells in Table 1 are normalized, and the mechanism modeling data in Table 3 are time-coded.

### 4.2. Evaluation Metrics

This study used the MSE, Mean Absolute Error (MAE) [29], and Relative Standard Error (RSE) as evaluation indicators to comprehensively measure the prediction performance of the model. The smaller the values of the three indicators, the closer the distance between the predicted value and the true value, and the better the prediction effect.

(34) $$MSE=\frac{1}{m}\sum_{I=1}^{M}{\left(y_{i}-{\hat{y}}_{i}\right)}^{2}$$

(35) $$MAE=\frac{1}{m}\sum_{i=1}^{m}\left|y_{i}-{\hat{y}}_{i}\right|$$

(36) $$RSE=\frac{s}{\bar{y}}\times 100\%$$

### 4.3. Mechanism–Data Fusion Prediction Model Analysis

In this study, data collected from a single wellhead on a particular day in a specific oilfield were divided into training, validation, and testing sets in a 7:1:2 ratio. The model was trained using the Adam optimizer with a learning rate ranging from 1 × 10^−4^ to 1 × 10^−3^ [30]. After performing correlation analysis, the selected oil well feature data were fed into the forecasting model. Meanwhile, time-encoded mechanistic model data were incorporated into the model’s loss function as additional constraints.

For comparative experiments, three prediction models—the Global–Local Branching Prediction Model, Autoformer, and DLinear—were evaluated under two conditions: with mechanistic model data and without mechanistic model data. All experiments were repeated three times to mitigate random fluctuations in the results.

As shown in Table 4, the proposed Global–Local Branching Prediction Model outperformed the other models in both scenarios, while the inclusion of mechanistic data generally yielded better forecasting accuracy compared with models without mechanistic data. For example, under the no-mechanistic-data condition, the proposed method achieved MSEs of 0.324, 0.326, and 0.325; MAEs of 0.1008, 0.101, and 0.1007; and RSEs of 12.808%, 12.838%, and 12.797%, respectively. Compared with the Autoformer model, the MSE values were reduced by 0.081, 0.074, and 0.074; the MAE values were reduced by 0.0172, 0.014, and 0.0153; and the RSE values were reduced by 1.49%, 1.371%, and 1.401%. Detailed comparative results are provided in Table 5.

**Figure 13:**
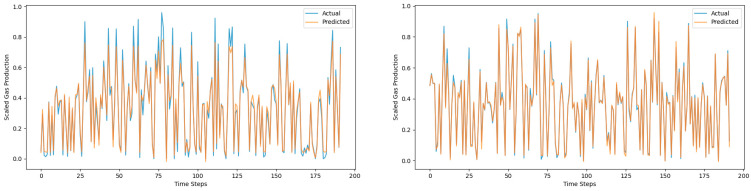
Global–Local network prediction results.

**Figure 14:**
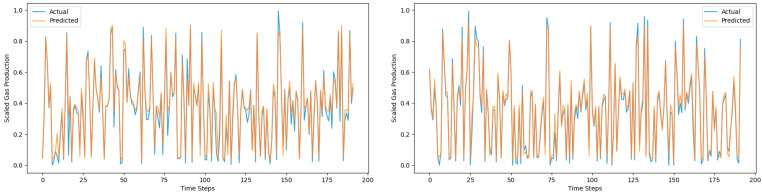
Autoformer prediction results.

**Figure 15:**
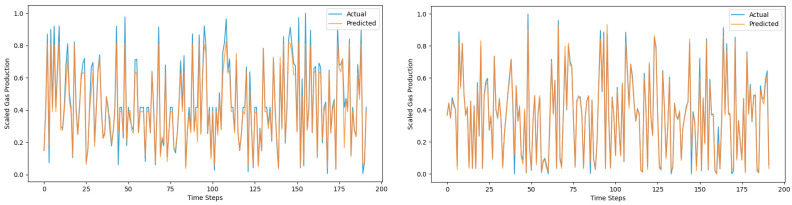
DLinear prediction results.

Figure 13, Figure 14 and Figure 15 are prediction comparison charts. The blue represents the actual data, the orange represents the predicted data, the right-sided figures show results with the mechanism data added, and the left-sided figures show results without the mechanism data added. It can be seen that the prediction effect of the Global–Local Branch Prediction Model is the best with or without the mechanism data, and the accuracy of the model prediction becomes better when the mechanism data are added.

The experimental results presented in this section validate the prediction model presented in Section 4 using the mechanistic model data in Section 3 and historical data. As described in the methodology, we constructed a mechanistic model based on the physical characteristics of the three-phase separator and output mechanistic data. The experimental results show that the prediction model presented in Section 4 successfully integrates the mechanistic data and historical data and outperforms existing methods in terms of prediction accuracy.

## 5. Conclusions

This paper first fused the test model data and actual production data for production prediction. A production prediction model that fuses mechanisms and data was designed to accurately describe the characteristics of oil well data from both the mechanism and reality perspectives, achieving more accurate prediction results. The Mechanism–Data Fusion Prediction Model architecture proposed in this paper has the following characteristics:

(1) This paper proposes a Mechanism–Data Fusion Prediction Model, which fuses two types of data into the time-series prediction model to achieve the multi-angle prediction of production. This is a new production prediction method and has achieved certain results.
(2) This paper also proposes a Global–Local Branch Prediction Model. By extracting global and local information from the input sequence, this model can effectively capture global information and integrate it with a module based on local attention, thereby significantly improving prediction accuracy.
(3) The experiments show that the Global–Local Branch Prediction Model is superior to other model algorithms, and the performance is also improved after the fusion of the mechanism model data.

## Figures and Tables

**Table 1:** Three-phase separator model parameters.

Parameter	Unit	Meaning
$Q_{1}$	$m^{3}/s$	Inlet volume flow
$Q_{2}$	$m^{3}/s$	Oil inlet volume flow
$Q_{3}$	mol/s	Inlet molar flow
$Q_{\mathrm{go}}$	mol/s	Outlet molar flow
$Q_{\mathrm{wo}}$	$m^{3}/s$	Outlet volume flow
$P_{\mathrm{wo}}$	$P_{a}$	Outlet pressure of water outlet valve
$P_{\mathrm{oo}}$	$P_{a}$	Oil outlet valve outlet pressure
$P_{\mathrm{go}}$	$P_{a}$	Outlet pressure of air outlet valve
$K_{i}$	/	Flow characteristic curve slope
$S_{1}$	$m^{2}$	Water outlet valve cross-sectional area
$S_{2}$	$m^{2}$	Oil outlet valve cross-sectional area
$S_{3}$	$m^{2}$	Exhaust valve cross-sectional area
$h_{1}$	m	Water chamber liquid level
$h_{2}$	m	Oil chamber liquid level
*H*	m	Weir plate height
V	$m^{3}$	Separator volume
P	$P_{a}$	Tank pressure
$A_{1}$	$m^{2}$	Oil–water interface cross-sectional area
$A_{2}$	$m^{2}$	Oil chamber cross-sectional area
T	K	Temperature in separator
$M_{w}$	/	Relative molecular weight
R	m	Separator radius
L	m	Separator length
$\rho_{1}$	${\mathrm{kg}/m}^{3}$	Water phase density
$\rho_{2}$	${\mathrm{kg}/m}^{3}$	Oil phase density
$\mu_{i}$	/	Valve opening

**Table 2:** Well data type.

Data Name	Unit
Gas production	m^3^
Chip catcher pressure	MPa
Chip catcher pressure	MPa
Separator upper pressure	MPa
Separator down pressure	MPa
Separator temperature	°C

**Table 3:** Mechanistic model data.

Data Name	Unit
Separator pressure	MPa
Separator level	MPa
Separator temperature	°C

**Table 4:** Experimental results.

	Global–Local Branching Prediction Model			Autoformer			DLinear		
	Mse	Mae	Rse	Mse	Mae	Rse	Mse	Mae	Rse
Organic Mechanism	0.0256	0.0895	11.773%	0.0383	0.1371	13.197%	0.0339	0.1002	12.336%
	0.0266	0.0917	11.872%	0.0346	0.1422	13.218%	0.0328	0.1001	12.296%
	0.0259	0.0923	11.932%	0.0352	0.1447	13.351%	0.0353	0.1011	12.380%
No Mechanism	0.0324	0.1008	12.808%	0.0405	0.1181	14.298%	0.0359	0.1023	13.801%
	0.0326	0.101	12.838%	0.0400	0.1153	14.209%	0.0364	0.1019	13.791%
	0.0325	0.1007	12.797%	0.0399	0.1164	14.198%	0.0368	0.1020	13.792%

**Table 5:** Comparison of experimental results.

	Global–Local Branch Prediction Model Prediction Results			Comparison of Prediction Results Between Global–Local Branch Prediction Model and Autoformer			Comparison of Prediction Results Between Global–Local Branch Prediction Model and DLinear		
	Mse	Mae	Rse	Mse	Mae	Rse	Mse	Mae	Rse
Organic Mechanism	0.0256	0.0895	11.773%	↓0.0127	↓0.0476	↓1.424%	↓0.0083	↓0.0107	↓0.563%
	0.0266	0.0917	11.872%	↓0.008	↓0.0505	↓1.346%	↓0.0062	↓0.0084	↓0.424%
	0.0259	0.0923	11.932%	↓0.0093	↓0.0524	↓1.419%	↓0.0094	↓0.0088	↓0.448%
No Mechanism	0.0324	0.1008	12.808%	↓0.0081	↓0.0173	↓1.49%	↓0.0035	↓0.0015	↓0.993%
	0.0326	0.101	12.838%	↓0.0074	↓0.0143	↓1.371%	↓0.0038	↓0.0009	↓0.953%
	0.0325	0.1007	12.797%	↓0.0074	↓0.0157	↓1.401%	↓0.0043	↓0.0013	↓0.995%

The arrows represent how much the Global-Local Branch Prediction Model has decreased compared to the prediction results of Autoformer and DLinear.

## Data Availability

The data are subject to third party restrictions.

## Abbreviations

The following abbreviations are used in this manuscript:

LSTM	Long Short-Term Memory
SVM	Support Vector Machine
ARIMA	Autoregressive Integrated Moving Average
PID	Proportional-Integral Derivative
SOTA	State Of The Art
MPC	Model Predictive Control
MSE	Mean Square Error
MAE	Mean Absolute Error
RSE	Relative Standard Error

## References

[1] Bratvold R.B., Bickel J.E., Lohne H.P. (2009). Value of information in the oil and gas industry: Past, present, and future. SPE Reserv. Eval. Eng..

[2] Rehman A., Zhu J.J., Segovia J., Anderson P.R. (2022). Assessment of deep learning and classical statistical methods on forecasting hourly natural gas demand at multiple sites in Spain. Energy.

[3] Wei B., Qiao R., Hou J., Wu Z., Sun J., Zhang Y., Qiang X., Zhao E. (2025). Multiphase production prediction of volume fracturing horizontal wells in tight oil reservoir during cyclic water injection. Phys. Fluids.

[4] Liu W., Liu W.D., Gu J. (2020). Forecasting oil production using ensemble empirical model decomposition based Long Short-Term Memory neural network. J. Pet. Sci. Eng..

[5] Qiao Y., Peng J., Ge L., Wang H. (2017). Application of PSO LS-SVM forecasting model in oil and gas production forecast. Proceedings of the 2017 IEEE 16th International Conference on Cognitive Informatics & Cognitive Computing (ICCI*CC).

[6] Rajni R., Banerjee T., Kumar P. (2024). Forecasting of renewable energy production in United States: An ARIMA based time series analysis. AIP Conf. Proc..

[7] Eshkalak M.O., Aybar U., Sepehrnoori K. (2014). An integrated reservoir model for unconventional resources, coupling pressure dependent phenomena. Proceedings of the SPE Eastern Regional Meeting.

[8] Ali A.A., Abdul-Majeed G.H., Al-Sarkhi A. (2024). Review of multiphase flow models in the petroleum engineering: Classifications, simulator types, and applications. Arab. J. Sci. Eng..

[9] Zhao X., Liu X., Yang Z., Wang F., Zhang Y., Liu G., Lin W. (2021). Experimental study on physical modeling of flow mechanism in volumetric fracturing of tight oil reservoir. Phys. Fluids.

[10] Jiang J., Yang J. (2018). Coupled fluid flow and geomechanics modeling of stress-sensitive production behavior in fractured shale gas reservoirs. Int. J. Rock Mech. Min. Sci..

[11] Sayda A.F., Taylor J.H. (2007). Modeling and control of three-phase gravilty separators in oil production facilities. Proceedings of the 2007 American Control Conference.

[12] Alzahra A.M., Najim Y., Dawood A. (2024). Three Phase Oil Separator Simulation Using CFD Analysis: A Review Study. Al-Rafidain Eng. J..

[13] Ahmed T., Makwashi N., Hameed M. (2017). A review of gravity three-phase separators. J. Emerg. Trends Eng. Appl. Sci..

[14] Ahmed T., Russell P.A., Makwashi N., Hamad F., Gooneratne S. (2020). Design and capital cost optimisation of three-phase gravity separators. Heliyon.

[15] Ghaffarkhah A., Shahrabi M.A., Moraveji M.K. (2018). 3D computational-fluid-dynamics modeling of horizontal three-phase separators: An approach for estimating the optimal dimensions. SPE Prod. Oper..

[16] Xu B., Shen J., Liu S., Su Q., Zhang J. (2020). Research and development of electro-hydraulic control valves oriented to industry 4.0: A review. Chin. J. Mech. Eng..

[17] Bu T., Mesa D., Brito-Parada P.R. (2024). Design strategies for miniaturised liquid–liquid separators—A critical review. Chem. Eng. J..

[18] Vaswani A., Shazeer N., Parmar N., Uszkoreit J., Jones L., Gomez A.N., Kaiser Ł., Polosukhin I. (2017). Attention is all you need. Adv. Neural Inf. Process. Syst..

[19] Xing S., Niu J., Ren T. (2023). GCFormer: Granger Causality based Attention Mechanism for Multivariate Time Series Anomaly Detection. Proceedings of the 2023 IEEE International Conference on Data Mining (ICDM).

[20] Li Y., Cai T., Zhang Y., Chen D., Dey D. (2022). What makes convolutional models great on long sequence modeling?. arXiv.

[21] Zhu Q., Zhang Y., Wang L., Zhong Y., Guan Q., Lu X., Zhang L., Li D. (2021). A global context-aware and batch-independent network for road extraction from VHR satellite imagery. ISPRS J. Photogramm. Remote Sens..

[22] Li Z., Liu F., Yang W., Peng S., Zhou J. (2021). A survey of convolutional neural networks: Analysis, applications, and prospects. IEEE Trans. Neural Netw. Learn. Syst..

[23] Mohammadi Foumani N., Miller L., Tan C.W., Webb G.I., Forestier G., Salehi M. (2024). Deep learning for time series classification and extrinsic regression: A current survey. ACM Comput. Surv..

[24] Yang Y., Peng Z., Zhang W., Meng G. (2019). Parameterised time-frequency analysis methods and their engineering applications: A review of recent advances. Mech. Syst. Signal Process..

[25] Gu A., Goel K., Ré C. (2021). Efficiently modeling long sequences with structured state spaces. arXiv.

[26] Venkatappareddy P., Culli J., Srivastava S., Lall B. (2021). A Legendre polynomial based activation function: An aid for modeling of max pooling. Digit. Signal Process..

[27] Kadambi A., de Melo C., Hsieh C.J., Srivastava M., Soatto S. (2023). Incorporating physics into data-driven computer vision. Nat. Mach. Intell..

[28] Duan J., Xiong J., Li Y., Ding W. (2024). Deep learning based multimodal biomedical data fusion: An overview and comparative review. Inf. Fusion.

[29] Frías-Paredes L., Mallor F., Gastón-Romeo M., León T. (2017). Assessing energy forecasting inaccuracy by simultaneously considering temporal and absolute errors. Energy Convers. Manag..

[30] Pérez M. An Investigation of ADAM: A Stochastic Optimization Method. Proceedings of the 39th International Conference on Machine Learning.

